# Beliefs and Practices of Patients with Diabetes toward the Use of Herbal Therapy

**DOI:** 10.3934/publichealth.2017.6.650

**Published:** 2018-01-04

**Authors:** Besher Gharaibeh, Loai Tawalbeh

**Affiliations:** 1 Faculty of Nursing, Jordan University of Science and Technology, Jordan; 2 Faculty of Nursing, Al-AlBayt University, Jordan

**Keywords:** beliefs, diabetes, herbal therapy, nursing, self-care

## Abstract

This study aimed to assess the prevalence of using herbal therapy and the beliefs toward the use of this type of therapy among patients with diabetes. It also aimed to identify the significant predictors of these beliefs and the factors that increase the likelihood of using herbal therapy. A descriptive cross-sectional design was used. A convenience sample comprised 310 patients with diabetes. Sixty-seven (21.6%) of the participants used herbal therapy. The mean beliefs score was 3.72 and ranged from (0–12). Linear regression showed that beliefs were significantly predicted by self-care, attending workshops, education level, and number of complications. The logistic regression showed that the lower the self-care and the higher the beliefs, the more likelihood the patient uses herbal therapy. Informing patient through individualized diabetes education influences the patient's beliefs and promotes self-care. This education program should target mainly those patients with low self-care, high number of complications, lower educational level and having more complications.

## 1. Introduction

Diabetes is a chronic disease that influences about 30 million people in the United States, or about 8% of the population [1]. In Jordan, diabetes is prevalent and is estimated to affect about 1.3 million or about 13% of the total population [2]. Self care is an essential part of managing diabetes and characterized by its challenging environment and its dynamic nature [1],[3]. Thus, addressing issues regarding diabetes self-care behaviors is important and should be done iteratively [4].

The use of herbal therapy to manage diabetes has gained more attention with more studies addressing this issue specially from medical and pharmaceutical perspectives [5]–[7]. The majority of these studies focused at investigating the effect of various plants on the level of blood glucose and glucose metabolism. American Diabetes Association [8] reported that there is abundance of research on the topic of herbal therapy; but there is still no consensus about its efficiency, and there is no proof indicating that herbal therapy helps in managing diabetes. Even though it is thought that these herbal supplements are generally safe and have very few adverse effects, some studies reported safety concerns regarding the extended use of certain herbal supplements and serious side effects such as hypoglycemia, medication interactions, and in some cases death [8],[9].

Despite the disagreement about the effectiveness of the herbal remedies and herbal therapy, about one fifth of the patients with diabetes are using these unconventional methods. This percentage tends to increase within certain ethnic groups [8]. In addition, those who use herbal therapy do not inform their physician about it [8]. In Jordan, one study [10] addressed the use of herbal therapy. The findings revealed that 46 plant species were used in herbal medicine to manage various diseases including diabetes. The authors noted that some of these plants used for curing diabetes are considered moderately unsafe or toxic.

The studies that explored the use of herbal medicine did not explain the reasons for doing so from patients' perspective. Instead, they focused on assessing the effect of the identified herbal therapy on glycemic control and the likelihood of diabetes complications [5]. Moreover, achieving glycemic control is multifaceted and depends not only on medications, but also on other factors such as lifestyle modification and self-care management [3],[11]. Related literature indicated that knowing the patient's attitudes and beliefs toward diabetes and its managements is essential for promoting adherence to self-care and reducing complications [12],[13]. Moreover, the ongoing process of self-care management should be built upon evidence-based standards and should consider the cultural influences to achieve the goals of self-care and to maintain a healthy lifestyle.

The American Diabetes Association (ADA) [8] reported that this type of therapy is more common in certain ethnicities than others, and that this therapy mostly disappeared in occidental societies. However, no explanation was provided for these findings. So, the aim of this study was to assess the beliefs and practices toward using herbal therapy from patients' perspective. It aids in shedding light on the factors that influence the patients to use herbal therapy despite the uncertainty of its efficiency. This study was guided by the conceptual framework (theory of reasoned action) developed by Ajzen and Fishbein [14] who postulated that beliefs precede and determine the actions.

## 2. Methods

### 2.1. Design

A cross-sectional descriptive design was used to determine the prevalence of using herbal therapy to identify the patients' beliefs toward the usefulness of herbal therapy among Jordanian patients with diabetes, and to identify the significant predictors of beliefs and the significant predictors of using herbal therapy.

### 2.2. Sample and Sampling Technique

A convenience sample of participants with type 1 and type 2 diabetes was used. The target population of the current study was all patients with diabetes in Jordan. The accessible population of the present study was all patients with diabetes referred to the selected governmental hospitals in Jordan. Eligibility criteria were: patients diagnosed with diabetes aged over 18 years; did not have cognitive impairment that was confirmed by patient's physicians; agreed to participate, and should be able to read and write Arabic.

G* power software [15] was used to compute the total sample size. Using an alpha level of 0.05, a power level of 0.95, an effect size of 0.15 for multiple regression analysis, and 9 predictors, the projected sample size was 166. To overcome the problem of attrition and missing or incomplete data, extra 40 participants were asked to participate to have at least 204 participants in the final sample size.

### 2.3. Setting

The sample was collected from central and major governmental hospitals located in various major cities in Jordan. The hospitals were operated by the Ministry of Health (MOH) and selected as the settings of the study. The usual total capacity for the selected hospitals ranged from 300–400 beds. Data were collected after approaching the patients during their visit to the outpatient endocrine, diabetes, or the cardiovascular clinics. Out-patient clinics include different specialities such as, internal medicine, cardiac, maternity, and vascular surgery. The endocrine, diabetes and cardiac clinic in each of the participating hospitals received approximately 100–150 patients with different health problems each day.

### 2.4. Data Collection Procedure

Once the ethical approval was obtained from the ethical research committee from a governmental University and from the institutional review board (IRB) of the MOH, a pilot testing was performed using ten participants who were not included in the final sample size. The result showed that there were no problems encountered during the process of data collection, coding, management and analysis. Self-reporting technique of whether or not the person has diabetes was used to identify those who fit the inclusion criteria. The patients, who met the inclusion criteria and formally agreed to participate, were provided with a brief description about the study and its purpose. Then, the participant received a questionnaire package. A cover letter containing a summary of the study, the participant's rights, and the researcher's contact information were included with the questionnaire packet. The cover letter also encouraged the potential participants to complete the questionnaire and return it as soon as possible to the Research Assistants. The estimated time to complete the questionnaire was 25 minutes. The data were collected between August and October, 2016.

### 2.5. Instrument

To measure the study variables, a questionnaire package was developed. This package contained three parts to measure beliefs and practices, self-care management, and demographic factors.

### 2.6. Beliefs Tool

The patients' belief was assessed using a self-developed questionnaire. This questionnaire had six questions related to the participants' beliefs about the impact of using herbal therapy on blood sugar and diabetes complications. These six questions were rated using scale where (0 = no, 1 = I do not know, 2 = yes). Summing the scores of the questions yielded a result that, with higher score, indicated more tendency/positive beliefs toward using herbal therapy. The possible scores ranged from 0–12. These questions were: Do you think herbal therapy help improve blood sugar? Do you think herbal therapy helps prevent diabetes complications? Do you think herbal therapy helps decrease the severity of diabetes symptoms? Do you think herbal therapy helps decrease how often you need a doctor? Do you think herbal therapy helps decrease how often you feel sick? And do you think herbal therapy helps improve you general health?

The internal consistency reliability for this measure was (Chronbach's alpha = 0.95). The practice was measured using yes-no question of whether or not the participant used herbal therapy to manage diabetes. In addition, the content validity index for the tool was 0.87 indicating that the tool was valid.

### 2.7. Diabetes Self-Management Scale

Self-care management was measured using the 40-item version of Diabetes Self-Management Scale (DSMS) [11]. A Likert scale with options that range from 0 = strongly disagree, to 5 = strongly agree was used. The possible total score ranges from 0–200 with a higher score indicating higher self-care. The 40- item DSMS was translated to Arabic because it is the native and first language in Jordan. The internal consistency reliability coefficient for the translated scale in this study was (Cronbach's alpha = 0.98).

A socio-demographic sheet was used to collect demographic data from the participants. This sheet also included questions that assess presence of support system by the family, the number of complications the patient has, and prior participation in self-care workshop (See table 1 for the variables that are measured as part of the socio-demographic factors). The translation and back translation for the questionnaire were performed by two doctoral prepared experts in nursing, one physician specialized in diabetes and two experts in both Arabic and English. No contradiction between the original and the translated tool was found. For suitable language use and cultural fitness, the translated tools were also evaluated by another Jordanian holding a doctoral degree in nursing.

### 2.8. Ethical Consideration

The study method was approved by the ethical research committee of the governmental University and by the IRB of the MOH. Written informed consent was attained from the participants who decided to take part in the study. The confidentiality of participant was achieved by writing a code number at the data collection sheet during the process of data collection and analysis. The participation was fully voluntary and participants were assured that their answers will be confidential. The participants had the right to withdraw from the study without any penalty. The participants received the required information regarding the estimated time and the contact information during the data collection. No harm or risk influenced the participants.

### 2.9. Data Analysis

Statistical Package of Social Science (SPSS) Version 22 was used to analyze the data. Descriptive statistics including mean, standard deviation (SD), frequency and percent were used to describe the sample characteristics and to determine the beliefs and practices, and the extent of using herbal therapy among the participants. Moreover, standardized multiple linear regression analysis was performed to identify the significant predictors of the beliefs about the usefullness of herbal therapy among patients with diabetes. Finally, logistic regression analysis was applied to determine the factors that increase the likelihood of using herbal therapy.

## 3. Results

### 3.1. Sample Characteristics

A convenience sample of patients with diabetes (N = 314) participated in the current study. Four cases were deleted from the analysis since they contained incomplete information, resulting in a final sample size of (N = 310). The results showed that almost 22% of the total sample used herbal therapy to treat diabetes. In addition, the mean beliefs score was 3.72 (SD = 1.66). The mean self-care score was 112.12 (SD = 53.73). The sample characteristics regarding the demographic variables and main study variables (beliefs and self-care) are presented in Table 1.

**Table 1.** Sample characteristics; mean (M); standard deviation (SD) and percent (%) for the patients with diabetes in Jordan (N = 310).

Variables	Range	M (SD)	N	%
Age (Years)	28.00–78.00	55.85 (12.12)	310	100
Duration of disease	01.00–43.00	08.85 (4.85.12)	310	100
Self care score	19–191	112.12(SD = 53.73)	310	100
beliefs score	0–4	1.24 (1.66)	310	100
Number of complications	0–9	2.53(1.70)	310	100
Gender				
*Male*			176	56.80
*Female*			134	43.20
Marital status				
*Married*			243	78.4
*Not married*			67	21.6
Do use herbal/traditional therapy				
*No*			243	87.4
*Yes*			67	21.6
Number of family members with diabetes			*125*	*40.3*
Type of diabetes				
*Type 1*			150	48.4
*Type 2*			169	51.6
Attending workshop				
*Yes*			179	57.7
*No*			131	43.3
Do you think herbal and traditional therapy improves blood sugar				
*No*			193	62.3
*I do not know*			40	12.9
*Yes*			77	24.8
Do you think herbal and traditional therapy prevents diabetes complications				
*No*			194	62.6
*I do not know*			41	13.2
*Yes*			75	24.2
Do you think herbal therapy help decrease the severity of diabetes symptoms				
*No*			101	32.6
*I do not know*			61	19.7
*Yes*			148	47.7
Do you think herbal therapy help decrease how often you need a doctor				
*No*			201	64.8
*I do not know*			40	12.9
*Yes*			69	22.3
Do you think herbal therapy help decrease how often you feel sick				
*No*			169	54.6
*I do not know*			53	17.1
*Yes*			88	28.3
Do you think herbal therapy help improve you general health				
*No*			103	33.2
*I do not know*			70	22.6
*Yes*			137	44.2

Further descriptive analyses were made to assess for prevalence of using herbal therapy within the two types of diabetes. Results showed that the prevalence of using herbal therapy among type 2 diabetes was significantly higher than that of type 1 diabetes (See Table 2).

**Table 2.** Using herbal and traditional therapy in the different types of diabetes.

Type of DM	Use herbal therapy		Total
	No	Yes	
Type 1	129	21	150
Type 2	114	46	160
Total	243	67	310

Chi square = 9.94; df = 1; *p* = 0.002.

Independent t-test was used to assess if there were statistically significant differences in the mean beliefs score in the dichotomous factors (type of diabetes, gender, and attending workshop, using herbal therapy). Results showed that there was statistically significant difference in the mean beliefs score between the types of DM, gender, attending workshop on diabetes, and using herbal therapy (See Table 3 for the results of the t-tests).

**Table 3.** Independent t-test to examine the difference in the beliefs scores within type of diabetes, gender, attending workshops, and using herbal therapy.

Variable name	Groups	M (SD)	t (308)	*P*
Type of diabetes	Type 1	0.906(1.4)	−3.49	0.001
	Type 2	1.55(1.78)		
Gender	Male	0.698(1.34)	−7.07	0.001
	Female	1.95(1.78)		
Attending workshop about diabetes	Yes	0.50(1.16)	10.6	0.001
	No	2.25(1.72)		
Use herbal therapy	Yes	3.3(1.31)	−15.15	0.001
	No	0.67(1.24)		

### 3.2. Predictors of the beliefs toward using herbal therapy

Standardized linear multiple regression analysis was performed to identify the significant predictors of the beliefs toward usefullness of herbal therapy among study participants. The results showed that the overall model, including all predictors of level of self-care, age, gender, duration of disease, educational level, number of family members with diabetes, type of diabetes, attending workshop about diabetes, and number of complication was statistically significant. Multiple *R = 0.655*, *R^2^ = 0.43*, adjusted *R^2^* was *0.41, F(9,300) = 24.8; p < 0.001*. This indicates that 41% of variance in beliefs score was explained by all predictors.

The results of the regression analysis showed that the beliefs score was significantly predicted by level of self-care, attending workshops, educational level, and number of complications. The squared semipartial correlation was calculated to assess the contributions of each individual significant predictor. The results showed that the unique contribution of the level of self-care was about 14.90%; for attending workshop was about 11.70%, for educational level was about 5.90%, and for the number of complications was about 4.80%. These results showed that the lower level of self-care, not attending workshops about diabetes, and the lower level of education and the more complications the patient suffers from, were associated with higher score of beliefs. Higher score of beliefs indicated higher possibility to use herbal therapy. Table 4 showed the standard linear multiple regression analysis.

**Table 4.** Standard linear multiple regression analysis to determine the significant predictors of beliefs toward using herbal therapy among patients with diabetes in Jordan (N = 310).

Variable	Unstandardized Coefficients	T	*P*-value	Semipartial (part) correlation
Self care	−0.010	−5.092	0.000*	−0.223
Attending workshop	−0.632	−3.205	0.001*	−0.140
Type of DM	0.007	0.044	0.965	0.002
Gender	−0.125	−0.664	0.507	−0.029
Age	0.006	0.836	0.404	0.037
Duration of diabetes	0.004	0.257	0.797	0.011
Educational level	−0.235	−3.041	0.003*	−0.133
Number of Complications	0.102	2.102	0.036*	0.092
Number of family members with diabetes	0.061	0.395	0.693	0.017

Dependent Variable: beliefs toward using herbal therapy. * *p* ≤ 0.05 level.

### 3.3. Predictors of using herbal therapy

Binomial logistic regression analysis was performed to identify the significant predictors of using herbal therapy. In this analysis, all the previously mentioned variables in multiple linear regression analysis were entered to the model in addition to the beliefs. The reason for adding beliefs to the model was the assumption that beliefs and attitudes precede actions [14]. Using the Chi-Square goodness of fit test, the null hypothesis that intercept and all coefficients are zero can be rejected. Nagelkerke R^2^ = 0.56; Cox & Snell's R^2^ = 0.36 indicating that about 36% chance of using herbal therapy is explained by the logistic model. Table 5 showed the results of logistic regression analysis.

**Table 5.** Logistic regression analysis using Wald Forward Method to determine the significant predictors of and the likelihood of using herbal therapy among patients with diabetes in Jordan (N = 310).

		B	S.E.	Wald	df	Sig	Exp(B)
Step 1^a^	beliefs	1.070	0.120	79.824	1	0.000*	2.916
	Constant	−3.452	0.369	87.635	1	0.000*	0.032
Step 2^b^	beliefs	0.871	0.129	45.763	1	0.000*	2.389
	Self care	−0.014	0.005	8.474	1	0.004*	0.986
	Constant	−1.769	0.616	8.246	1	0.004*	0.171

a. Variable(s) entered on step 1: beliefs; b. Variable(s) entered on step 2: self care; * *p* ≤ 0.05 level.

Using the Wald Forward Method, results showed that only beliefs and self-care significantly influenced the odds of using herbal therapy. Controlling for beliefs, the results showed that a 1 point higher score in the self-care, the odds of using herbs decrease by (0.98). Meanwhile, controlling for self-care, a one point increase in the beliefs score increases the odds of using herbal therapy by 2.39 times. In addition, calculating the critical value showed that if the patient scored higher than 126.4 on the self-care test, the patient will not use herbal therapy. Regarding the beliefs, the patient will use herbal therapy if he scores 6.09 or higher on the beliefs test.

## 4. Discussion

This study aimed to assess the prevalence of using herbal therapy among patients with diabetes, and their beliefs toward the usefulness of this type of therapy. It also aimed to identify the significant predictors of these beliefs and the factors that increase the likelihood of using herbal therapy. Searching the available literature yielded no similar studies that can provide clarification for most of our findings.

American diabetes Association published on yearly bases updates standards of medical care in diabetes to provide the clinicians with guidelines to help in managing their patients [16]. These guidelines include descriptions of pharmacological approaches necessary to achieve glycemic treatment. This glycemic treatment depends on complex protocol that incorporates the use of antihyperglycemic tablets and insulin injections. In addition, Diabetes Self Care Management (DSCM) is another important approach that helps manage diabetes through the performance of self-care activities [17]. Shrivastava et al. [12] study addressed the relationship between DSCM and glycemic control and concluded that glycemic control cannot be achieved without performing the DSCM activities.

Even though the evidence for its efficiency is not definitive, patients with diabetes continue to seek herbal therapy thinking that this behavior can control their blood sugar and manage their diabetes [8],[18]. Moreover, the majority of those who use herbal therapy do not inform their physicians about that [19]. The continuing use of this debatable approach of treatment stimulated an ongoing research on the issue of using herbal therapy in managing diabetes [5],[7].

This study demonstrated that the prevalence of using herbal therapy among Jordanian patients with diabetes is comparable to the reported ratios by ADA [8]. Some researchers [20] stated that the use of herbal therapy is a mainstay in the developing countries. Jordan is considered as one of the developing countries and has a substantial rate of poverty of 14.4% [21]. The lack of structured diabetes education may have an influence on the use of herbal therapy. This is congruent with the ADA reports [8] which showed that education by health care professional is essential to prevent the misuse of herbal therapy. Further research about the impact of structured education on beliefs and using alternative methods of therapy is needed.

The beliefs about the usefulness of herbal therapy were significantly predicted by the level of self-care, attending workshops, level of education, and number of complications. The beliefs were in favor of using herbal therapy if the patient had low level of self-care, did not attend diabetes workshops, had low level of education, or had more complications. For people with diabetes, some studies found that there is an association between knowledge, beliefs, and self-care [12],[22]. Attending workshops and the level of education are thought to improve the patient's knowledge, which is considered as prerequisite for performing appropriate self-care [3]. This relationship between self-care and beliefs is also reasonable considering that those who care for themselves properly recognize not to seek unproven methods of care. Moreover, in the current study, those with low number of complications had lower beliefs about the usefulness of herbal therapy, which may be attributed to that patient with high self-care had low number of complications [11]. The influence of high level of education on the beliefs toward the usefulness of herbal therapy can be intuitively justified by the fact that those with high education tend not to believe in the effectiveness of the traditional therapy.

Meanwhile, using herbal therapy as a behavior was significantly predicted by the beliefs and the level of self-care as indicated by logistic regression analysis, where low beliefs and high self-care levels reduced the likelihood of using herbal therapy. These findings are congruent with another study [12] that reported that adhering to self-care was dependent on the level of beliefs and the level of knowledge of patients with diabetes. In addition, theory of reasoned action [14] showed that behavior is influenced by both personal and social attitudes and beliefs. Considering that the behavior of using herbal therapy was found to be customary in the Jordanian population, we might conclude that both personal and social attitudes and beliefs have contributed to the event of using herbal therapy.

## 5. Conclusion

The discussion about using herbal therapy to manage diabetes in literature is abundant. Many plants were identified to affect blood glucose level and glucose metabolism, but there is no consensus about the efficacy of using herbal therapy to manage diabetes. Although the use of herbal therapy is neither proven nor recommended by the standards of diabetes care, the use of herbal therapy is not uncommon among patients with diabetes. This study examined the psychological perspective of this therapy and showed that the beliefs played a major role in predicting the use of herbs. Moreover, the study demonstrated that the likelihood of using herbal therapy is influenced by receiving education about diabetes. These findings emphasize the importance of providing comprehensive diabetes education, promoting self-care, and managing complications as those accomplishments reduce the possibility of the patients to seek unproven and unorthodoxed methods to manage their diabetes that may deteriorate there conditions.

The findings of this study emphasize the need to conduct individualized evaluation about patient's beliefs toward using alternative therapies. This evaluation can provide baseline for the individualized teaching that covers individual educational needs. These needs can include addressing the alternative herbal therapy commonly used within the context of the cultural or ethnical group the patient belongs to or influenced by. Moreover, this study demonstrated that the informed patient tends to have proper health-related beliefs which can influence health-related behavior. So, structured educational programs that consider patient characteristics and beliefs can play an important role in improving patient adherence to the proper therapy [23],[24]. Finally, this study has built a reliable, simple, and short tool to assess the beliefs toward using herbal therapy which can be helpful in identifying the beliefs and building an effective educational program. The external validity of the current findings might be influenced by the use of cross-sectional design and the convenient sampling technique. Future studies are recommended to explore the effect of specific educational programs on the belief and use of herbal therapy. Further research studies are recommended to explore new variables that could influence the belief and use of herbal therapy
